# Supplementary material for: MetaRibo-Seq measures translation in microbiomes
Source: Nat Commun. 2020 Jun 29;11:3268. doi: 10.1038/s41467-020-17081-z (PMC7324362; doi:10.1038/s41467-020-17081-z)
Supplement: Supplementary file 10 — Supplementary Data 7 [file 41467_2020_17081_MOESM10_ESM.zip › File2/Confidence_High_Taxonomy/313441_out.krona.html]

Javascript must be enabled to view this page.

members
magnitude
magnitudeUnassigned
count
unassigned
taxon
rank

313441\_out

3

2
3
superkingdom

1239
3
phylum

186801
3
class

186802
3
order

family
186803
3

207244
1
genus

1

SRS104636\_contig\_number\_22816
649756
species

2
841
genus

2
166486

SRS054956\_contig\_number\_3504SRS893380\_contig\_number\_contig-100\_1206.1207
species
